# Efficacy of EGFR Inhibition Is Modulated by Model, Sex, Genetic Background and Diet: Implications for Preclinical Cancer Prevention and Therapy Trials

**DOI:** 10.1371/journal.pone.0039552

**Published:** 2012-06-25

**Authors:** Erica S. Rinella, David W. Threadgill

**Affiliations:** 1 Department of Genetics, Curriculum in Genetics and Molecular Biology, Lineberger Cancer Center, Center for Gastrointestinal Biology and Disease, and Carolina Center for Genome Sciences, University of North Carolina, Chapel Hill, North Carolina, United States of America; 2 Department of Genetics, North Carolina State University, Raleigh, North Carolina, United States of America; Mizoram University, India

## Abstract

Molecule-targeted therapies are being widely developed and deployed, but they are frequently less effective in clinical trials than predicted based upon preclinical studies. Frequently, only a single model or genetic background is utilized using diets that are not relevant to that consumed by most cancer patients, which may contribute to the lack of predictability of many preclinical therapeutic studies. Inhibition of epidermal growth factor receptor (EGFR) in colorectal cancer was used to investigate potential causes for low predictive values of many preclinical studies. The efficacy of the small molecule EGFR inhibitor AG1478 was evaluated using two mouse models, *Apc^Min/+^* and azoxymethane (AOM), both sexes on three genetic backgrounds, C57BL/6J (B6) and A/J (A) inbred strains and AB6F1 hybrids, and two diets, standard chow (STD) or Western-style diet (WD). AG1478 has significant anti-tumor activity in the B6-*Apc^Min/+^* model with STD but only moderately on the WD and in the AOM model on an A background with a WD but not STD. On the F1 hybrid background AG1478 is effective in the *Apc^Min/+^* model with either STD or WD, but has only moderate efficacy in the AOM model with either diet. Sex differences were also observed. Unexpectedly, the level of liver EGFR phosphorylation inhibition by AG1478 was not positively correlated with inhibition of tumor growth in the AOM model. Model-dependent interactions between genetic background and diet can dramatically impact preclinical results, and indicate that low predictive values of preclinical studies can be attributed to study designs that do not account for the heterogeneous patient population or the diets they consume. Better-designed preclinical studies should lead to more accurate predictions of therapeutic response in the clinic.

## Introduction

While many new promising therapies are being approved for cancer treatment, it is becoming clear that their efficacy in clinical trials is not reflective of that predicted in preclinical studies. An analysis of published Phase 1 clinical trials from 1991–2002 revealed that only 3.8% of patients showed a significant clinical response [1]. Animal models used in preclinical studies to guide clinical trials have been useful in predicting targets and elucidating their mechanisms of action, but these models are rarely designed to incorporate heterogeneous factors faced in human clinical trials, such as genetics and diet, which likely contribute to the inconsistencies observed when moving a drug from preclinical to clinical status.

Among the most prevalent cancers, colorectal cancer (CRC) has the lowest Phase I response rate at 1.3% [1], necessitating an investigation into how preclinical studies can be improved. A target of significant interest over the last ten years, often touted as the prototype for molecule-targeted therapies [2], is the epidermal growth factor receptor (EGFR) whose signaling is deregulated in up to 50% of CRCs [3]. Numerous therapeutic agents have been developed to target EGFR with the hope that they would be potent CRC therapeutics. These therapies include small molecule inhibitors that target the kinase domain and blocking antibodies against the ligand-binding domain.

Mouse models developed to study the etiology of CRC are frequently used in preclinical studies. The most widely used models are the *Apc^Min/+^* and azoxymethane (AOM)-treated mouse models [4]–[7]. Previous studies have shown that *Apc^Min/+^* tumors display increased EGFR activity, and that treatment with small molecule EGFR inhibitors results in a significant decrease in tumor incidence in these mice [8]–[10]. In addition, though preclinical studies have yet to report efficacy of EGFR-inhibition in the AOM model, AOM-induced tumors show increased EGFR activity [11], [12], therefore it is reasonable to hypothesize that this model will also respond to EGFR-targeted treatment. Although many preclinical studies have provided evidence for the potential efficacy of EGFR-targeted treatment of CRC, these studies have not accurately predicted the poor response observed in the clinic. One possible issue with preclinical trials is the oversimplification of modeling human trials in mice.

Diet is rarely considered in the design of preclinical therapeutic studies. For example, Western-style diets commonly consumed in North America and Europe are characterized by high levels of fat and low amounts of fruits and vegetables, factors not considered when performing preclinical studies. Studies have linked Western-style diets to increased cancer incidence, including CRC. Modeling the Western-style diet in mice, with increased fat, decreased Vitamin D, and decreased calcium, results in increased hyperproliferation in pancreatic, prostate and mammary epithelial cells [13]. The same diet induces hyperproliferation in intestinal epithelial cells of wild-type C57BL/6 (B6) mice [14]. Further modification of the diet to include decreased levels of folate and other nutrients essential for DNA methylation induces adenomas and carcinomas in mice [14], [15].

In addition to the potential for diet to influence tumorigenesis, it is also evident that genetic background is important. As with human CRC, susceptibility to AOM-induced tumors is dependent on genetic background [16]–[18]. A/J (A) mice, for example, are highly susceptible to AOM-induced colon tumors whereas B6 mice are relatively resistant. Likewise, tumor incidence in the *Apc^Min/+^* model is greatly dependent on mouse strain, with B6 mice bearing significantly more tumors compared to F1 mice with less susceptible strains like AKR/J and A [19], [20].

With the knowledge that genetic background and diet differentially influence tumor incidence, it is reasonable to expect, albeit not yet tested, that they may also be important factors contributing to the variability of EGFR-targeted therapies. To determine the importance of experimental conditions on preclinical studies, we investigated the efficacy of the small molecule EGFR inhibitor AG1478 using different mouse models, diets and genetic backgrounds.

## Materials and Methods

### Ethics Statement

All experiments were approved by the UNC Institutional Animal Care and Use Committee (protocol #07-069.0).

### Experimental Design

**Figure 1:**
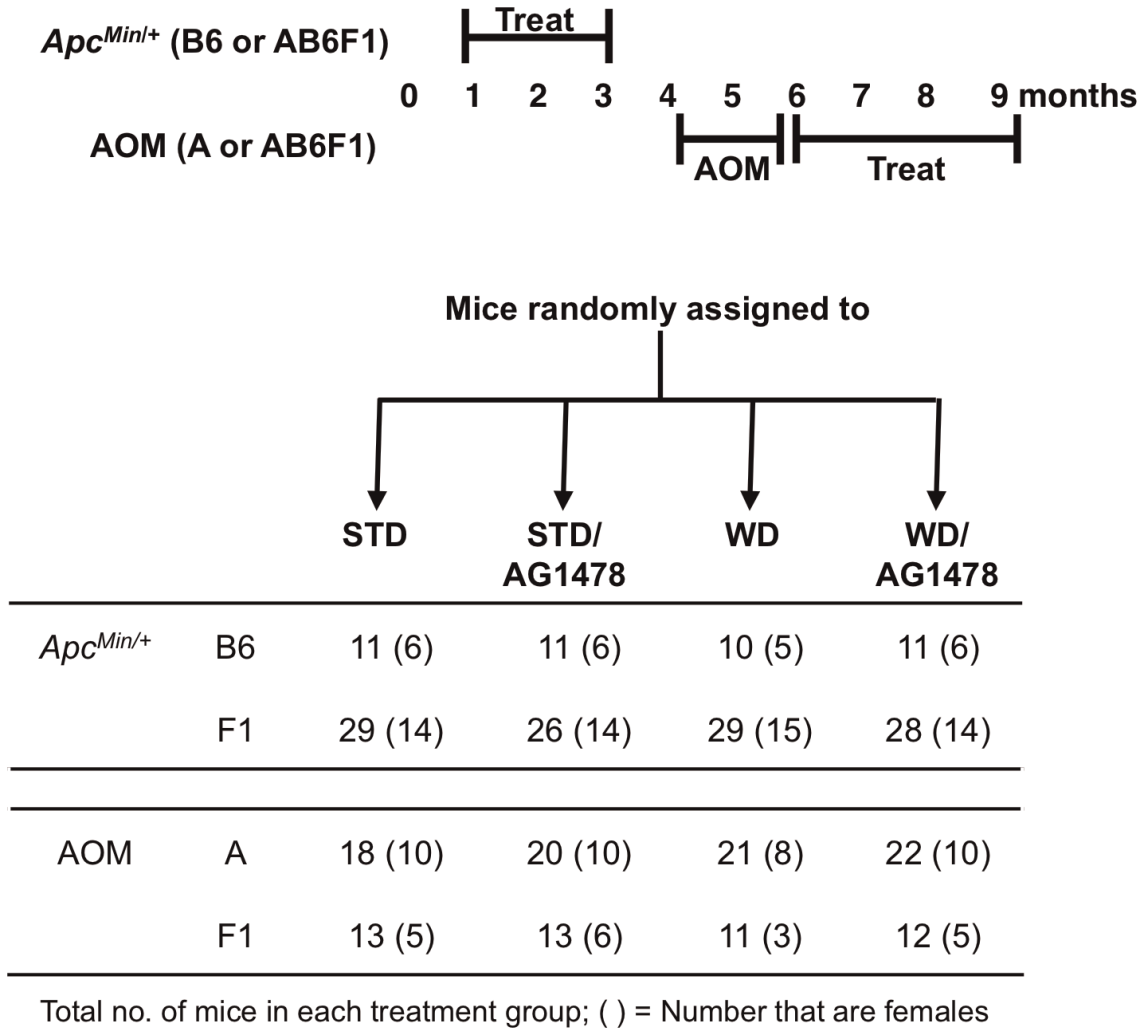
Experimental design. At weaning (*Apc^Min/+^)* or 1 week following the last carcinogen treatment (AOM) mice were randomly assigned to a Standard (STD) or Western-style (WD) base diet, with or without small-molecule EGFR inhibitor AG1478. The total number of mice per model/strain (N) is displayed in the table below the diagram with the number of females in parentheses.

Equal numbers of male and female inbred strain “A” mice were obtained from The Jackson Laboratory and maintained on 5058 mouse chow (LabDiet). Additionally, A X C57BL/6 F1 (AB6F1) hybrid mice were generated in house. At four months of age mice were injected weekly with AOM (10 mg/kg body weight) for six weeks (Figure 1). One week prior to the first AOM injection, mice were moved to a standard diet (STD; AIN-93G from Research Diets), then one week after the last AOM injection, mice were randomly assigned to one of two diets, STD or Western (WD; D12079B from Research Diets) (Table 1), with or without 144 mg/kg chow of EGFR-inhibitor AG1478 (LC Labs). WD is nutritionally matched with the STD except for increased fat and reduced fiber, calcium and vitamin D, more consistent with the diets consumed by people in North America. Mice were maintained on their respective diet for 12 weeks before being euthanized with CO_2_. Colons were dissected, flushed with phosphate-buffered saline (PBS), splayed on bibulous paper and scored for tumor number, diameter and location (in mm from distal end).

**Table 1:** Comparison of diet compositions.

	Standard Diet	Western Diet
**Calories (kcal/g)**	4	4.7
**Fat (kcal%)**	16	41
**Fat type**	Soybean oil	Milk, corn oil
**Carbohydrates (kcal%)**	64	43
**Sucrose (kcal%)**	10	29
**Protein (kcal%)**	20	17
**Cholesterol (%)**	0	0.21
**Fiber (%)**	5	2
**Calcium (g/kg)**	5	0.5
**Vitamin D (IU/kg)**	1000	100

Wild-type C57BL/6 (“B6”) and A females were also mated to B6-*Apc^Min/+^* males to generate B6-*Apc^Min/+^* inbred and AB6F1*-Apc^Min/+^* hybrids, respectively, and progeny containing the *Apc^Min^* allele were weaned at 3 weeks of age and assigned to STD or WD, with or without AG1478 (Figure 1). After 9 weeks of treatment mice were euthanized and the entire intestinal tract was flushed with PBS and splayed on bibulous paper. The intestine was divided into 4 sections: small intestine (duodenum, jejunum, and ileum) and colon.

General health of the mice was monitored by appearance (mobility and ability to groom) and body weights measured biweekly for the AOM model and weekly for the *Apc^Min/+^* model (data not shown as only genetic-background and diet, but not treatment or model, affected body weight). All mice were housed in an AAALAC approved-facility and provided chow and water *ad libitum*. Mice were kept specific pathogen free.

### Western Blot Analysis for EGFR Signal

Five minutes prior to euthanasia mice were injected with 10 µl/g body weight of 5 mM phosphatase inhibitor sodium orthovanadate (activated with 50 mM hydrogen peroxide 15 minutes prior to use) [21]. After euthanasia, livers were dissected and snap frozen in liquid nitrogen. Total protein was extracted with lysis buffer containing 10 mM Tris-HCl (pH 8), 100 mM NaCl, 1 mM EDTA, 1 mM EGTA, 1 mM NaF, 1% NP-40, 10% Glycerol, 0.1% SDS, 0.5% Sodium deoxycholate, 1 mM PMSF, 10 µg/ml leupeptin, 10 µg/ml aprotinin, 1 mM Na_3_VO_4_, and 1 mM NaF. A Bradford protein assay was used to determine protein concentration (Bio-Rad).

Thirty µg of liver protein was loaded onto 4–15% gradient Tris-HCl gels (Bio-Rad). After transfer to PVDF membranes for one hour, membranes were blocked for one hour in 5% BSA/TBS (0.1% Tween) for rabbit anti-pEGFR 1068 antibody (Cell Signaling) or 5% milk/TBST for sheep anti-EGFR (Millipore) and mouse anti-ACTB (Sigma) antibodies. Membranes were incubated with primary antibody overnight at 4°C. Antibodies against pEGFR were diluted 1∶2,000 in 1% BSA/TBST, EGFR diluted 1∶5,000 and ACTB diluted 1∶10,000 in 5% milk/TBST. After washing, membranes were incubated with secondary antibodies for one hour at room temperature. Secondary antibodies (Jackson ImmunoResearch) were diluted 1∶20,000 in the same solution as the primary antibody. SuperSignal West Dura Extended Duration ECL (Pierce) was used for visualization of proteins. A minimum of 4 replicates (2 per sex) were performed per treatment and model/strain.

### Statistical Analysis

Summary Table 2 was constructed using p-values from Mann-Whitney analysis (Prism 4.0 (GraphPad Software, Inc.)) of all possible pair-wise comparisons within each model/strain and treatment for association with tumor number or size (dependent variables). Sex was not a factor in this summary analysis. For *Apc^Min^* ^/+^ mice, p-values were corrected for 12 comparisons and p-values for AOM mice were corrected for 6 (half as many because tumors only occurred in the colon in this model). This analysis was done for tumor number and tumor size separately. Down arrows in the table indicate a corrected p-value of <0.05, while “trend” indicates an uncorrected p-value of <0.05 that rises above 0.05 after correction.

**Table 2:** Summary of pair-wise observations of AG1478 inhibitor effect on tumor growth.

		SD	SD	WD	WD
Strain	Model	Tumor Number	Tumor Size	Tumor Number	Tumor Size
B6	*Apc^Min/+^*	↓	–	trend	↓
ABF1	*Apc^Min/+^*	↓	↓	↓	↓
A	AOM	–	trend	–	–
ABF1	AOM	trend	–	–	–

SD standard diet; WD, western diet; ↓ corrected p<0.05; trend, uncorrected p<0.05 but corrected p>0.05.

Linear mixed models (SPSS v. 20) were performed separately for the *Apc^Min^* ^/+^ and AOM CRC mouse models to assess the impact of diet (STD vs WD), AG1478 treatment (“−“ no treatment vs “+” treatment), and sex on tumor number and size. Models were evaluated using mouse strain (B6 vs F1 for *Apc^Min^* ^/+^ and A vs F for AOM mice) as a random effect and diet, AG1478 and sex as fixed effects. Tests of all possible interactions between the fixed effects were also evaluated. Dependent variables were tumor number or tumor size. For the *Apc^Min^* ^/+^ model, small intestinal and colonic tumors were evaluated separately.

For pEGFR signal analysis, ImageJ open source software (rsbweb.nih.gov/ij/) was used to estimate the density of the bands on the western blots. For each individual blot, EGFR and pEGFR bands were normalized to the loading control (β-actin) that was run in the same lane. pEGFR was then calculated as a percentage of total EGFR for each lane. Finally, all treatments (STD/+AG1478, WD/−AG1478 and WD/+AG1478) were normalized to untreated (STD/−AG1478) for each blot. Mouse models/strains and sex were run on separate blots. A minimum of 4 replicates (2 per sex) were run per treatment and model/strain. Linear mixed models were performed to assess the association of diet, sex, AG1478 (fixed effects) and strain (random effect) with pEGFR signal. The dependent variable was the normalized pEGFR signal. Bar graphs were constructed using Prism 4.0 (GraphPad Software, Inc.).

## Results

### Western-style Diet Increases Tumor Growth in *Apc^Min/+^* Mice

**Figure 2:**
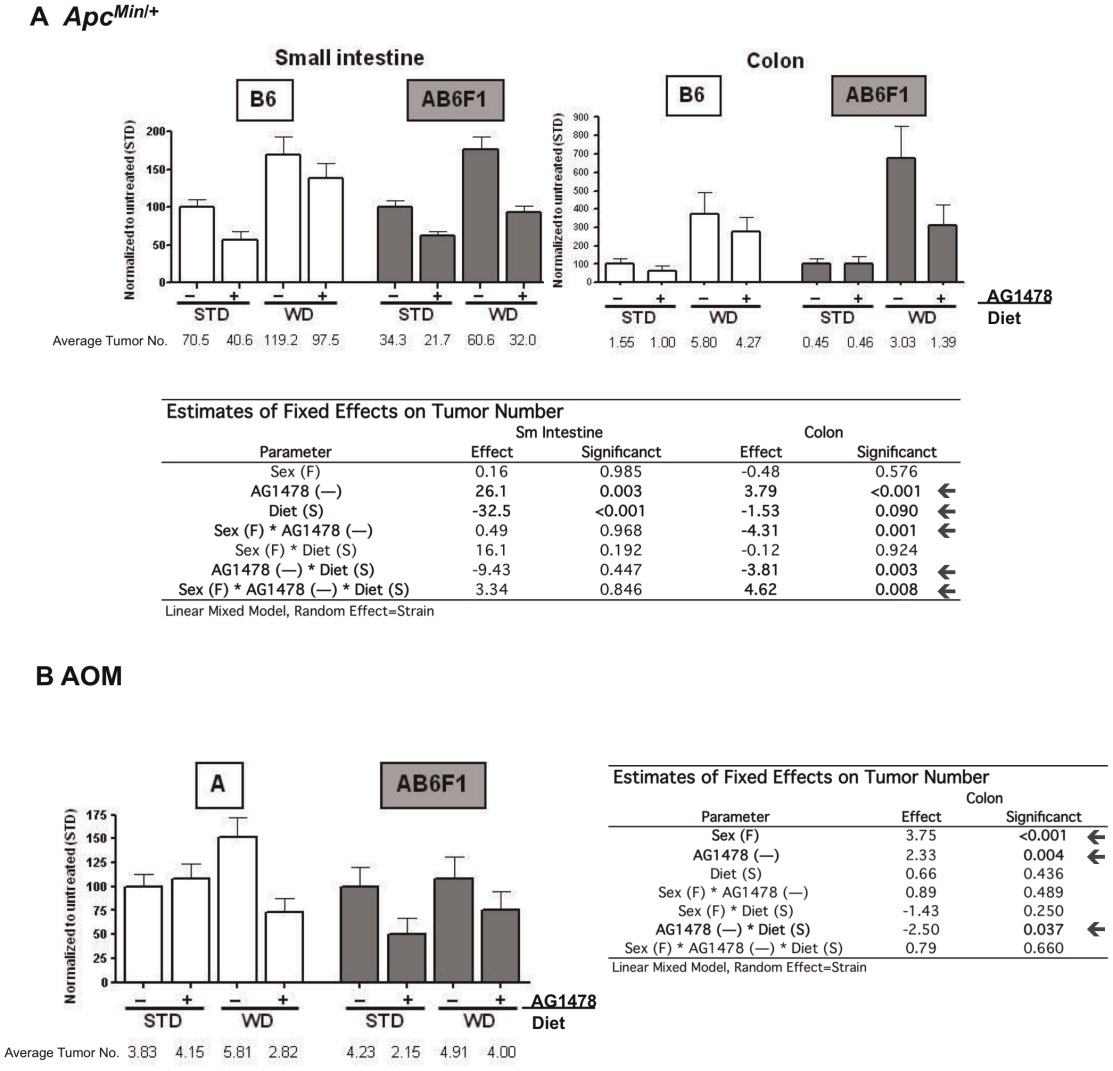
Effect of diet, sex and strain on AG1478-mediated tumor number reduction. A) *Apc^Min^* ^/+^ small intestinal and colonic tumor number as a percentage of STD without AG1478 treatment for the B6 inbred and AB6F1 (shaded) strains; and B) AOM (A and AB6F1) colonic tumor number expressed as a percent of STD treatment. The raw values for average number of tumors/mouse are displayed below each graph. STD = standard diet, WD = western diet, “+”  =  AG1478 treatment and “−“  =  no AG1478 treatment. Standard Error of the Mean bars are shown for each treatment. Below bar graphs are estimated effects and significance based on linear mixed models.

**Figure 3:**
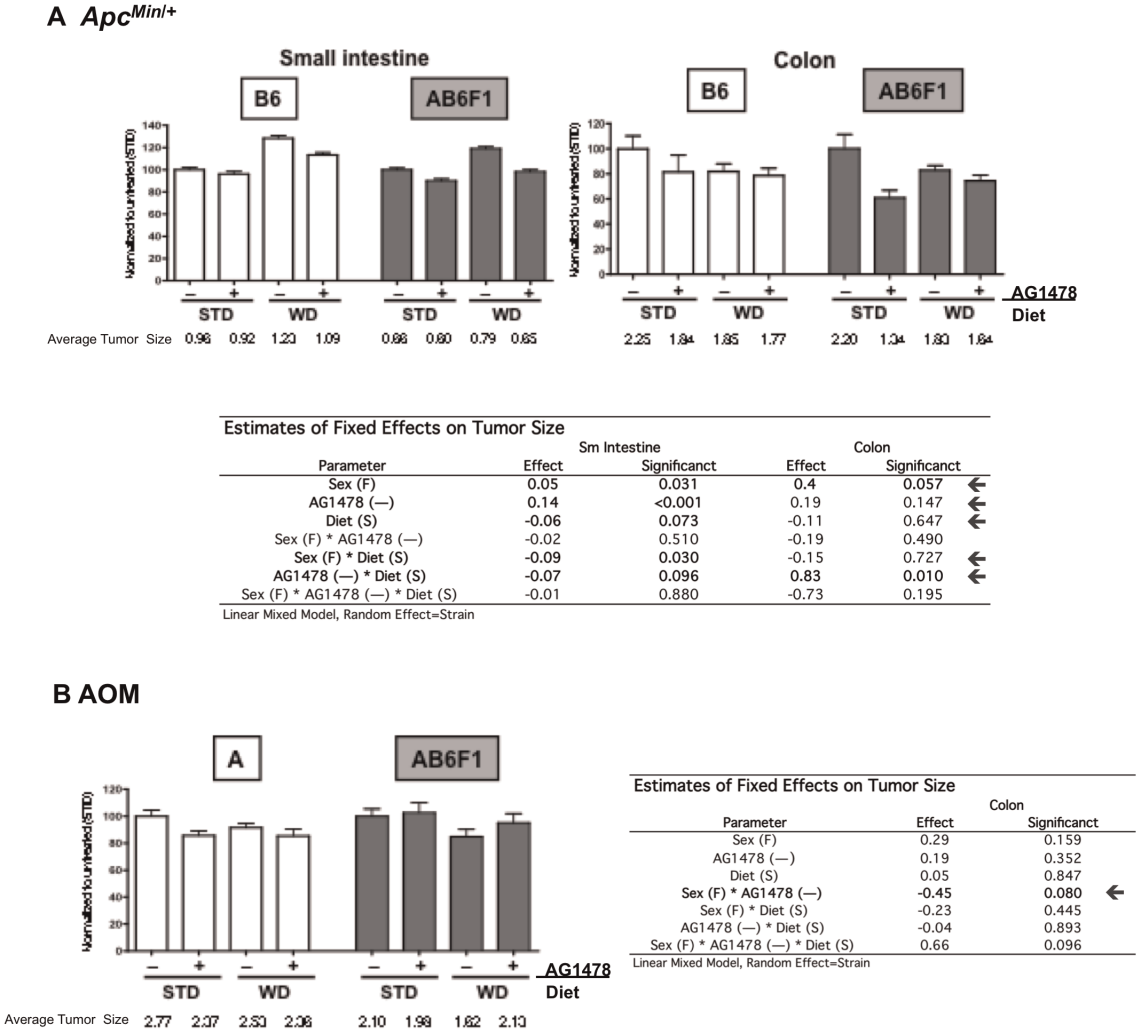
Effect of diet, sex and strain on AG1478-mediated tumor size(mm) reduction. A) *Apc^Min^* ^/+^ small intestinal and colonic tumor size as a percentage of STD without AG1478 treatment for the B6 inbred and AB6F1 (shaded) strains; and B) AOM (A and AB6F1) colonic tumor size expressed as a percent of STD treatment. The raw values for average size of tumors/mouse are displayed below each graph. STD = standard diet, WD = western diet, “+”  =  AG1478 treatment and “−“  =  no AG1478 treatment. Standard Error of the Mean bars are shown for each treatment. Below bar graphs are estimated effects and significance based on linear mixed models.

Consistent with previous reports, WD is associated with increased tumor number compared to a STD in *Apc^Min/+^* mice (Figure 2A; p<0.001 in small intestine and p = 0.090 in colon). WD was also moderately associated with increased size of small intestinal tumors in the *Apc^Min/+^* model (Figure 3A; p = 0.073). WD was not, however, associated with a change in tumor number or size in the AOM mouse model.

### Efficacy of EGFR Inhibition on Tumor Growth is Variable Across Diet, Model, Sex and Strain

Evaluation of the efficacy of EGFR inhibition on tumor growth showed variable results dependent on diet, model and genetic background. Table 2 shows general pair-wise observations of tumor number and size for each CRC mouse model, *Apc^Min/+^* and AOM. In addition to differential response observed between mouse models, we observe differences in response EGFR inhibitor AG1478 within each model according to strain (inbred vs F1). *Apc^Min/+^* mice on the F1 genetic background had the most favorable overall response to AG1478 with a decrease in tumor number and size on both the STD and WD diets. On the other hand, AOM mice on the F1 genetic background had the least favorable response to AG1478 with no significant decrease in tumor number or size across both diets. Only “trends” towards significance were observed for some of the models/strains as the pair-wise p-value rose above 0.05 after correction for multiple comparisons.

Linear mixed model results for tumor number (Figure 2) and tumor size (Figure 3) in the *Apc^Min/+^* and AOM models provide a more detailed assessment of the affect of diet, strain and sex on EGFR inhibition of tumor growth. Using strain as the random effect, we confirm previous reports [8]–[10] that pharmacologic inhibition of EGFR decreases small intestinal tumor number and size in the *Apc^Min/+^* mice (Figure 2A, p = 0.003 and Figure 3A, p<0.001, respectively). AG1478 is also associated with decreased colonic tumor number (Figure 2A, p<0.001). An interaction between AG1478 treatment and diet for this model, while controlling for strain, is observed in association with colonic tumor number and size (p = 0.003 and p = 0.010, respectively).

AG1478 treatment was also associated with decreased colonic tumor number in AOM mice (small intestinal tumors are not observed in this mouse model) (Figure 2B, p = 0.004). Similar to the *Apc^Min/+^* mice, AG1478 treatment and diet interact to impact colonic tumor number (but not size) in AOM mice (p = 0.037).

In addition to diet and AG1478 treatment, sex was included as a fixed effect when assessing tumor number and size in each model (with strain as the random effect). AOM females have a higher incidence of colonic tumors (Figure 2B, p<0.001). Additionally, *Apc^Min/+^* females have larger small intestinal and colonic tumors compared to males (Figure 3A, p = 0.031 and p = 0.057, respectively). An interaction between sex and AG1478 treatment is observed in association with colonic tumor number in *Apc^Min/+^* (Figure 2A, p = 0.001) and moderately with AOM tumor size (Figure 3B, p = 0.080). An interaction between all fixed effects (diet, strain and AG1478) is observed in association with colonic tumor number in *Apc^Min/+^* mice (Figure 2A, p = 0.008). Finally, sex and diet interact in association to affect small intestinal tumor size in *Apc^Min/+^* mice (Figure 3A, p = 0.030).

### Impact of AG1478 on EGFR Signaling is Dependent on Mouse Model and Diet

To evaluate the level of EGFR inhibition achieved by AG1478 under different experimental conditions, reduction in phosphorylated EGFR (pEGFR) was measured in liver lysates; there was insufficient material to directly measure levels in tumors. Analyses were performed by sex since sex-dependent effects were observed in the tumor growth analyses (Figures 2 and 3). Signal intensities were normalized to a loading control run in the same lane. pEGFR/EGFR was calculated for each lane and the pEGFR/EGFR ratios for STD/+AG1478, WD/−AG1478 and WD/+AG1478 were normalized to the STD untreated (no AG1478) pEGFR/EGFR ratio on the corresponding gel. This normalized ratio, “pEGFR signal”, was used as the dependent variable in subsequent linear mixed models.

**Figure 4:**
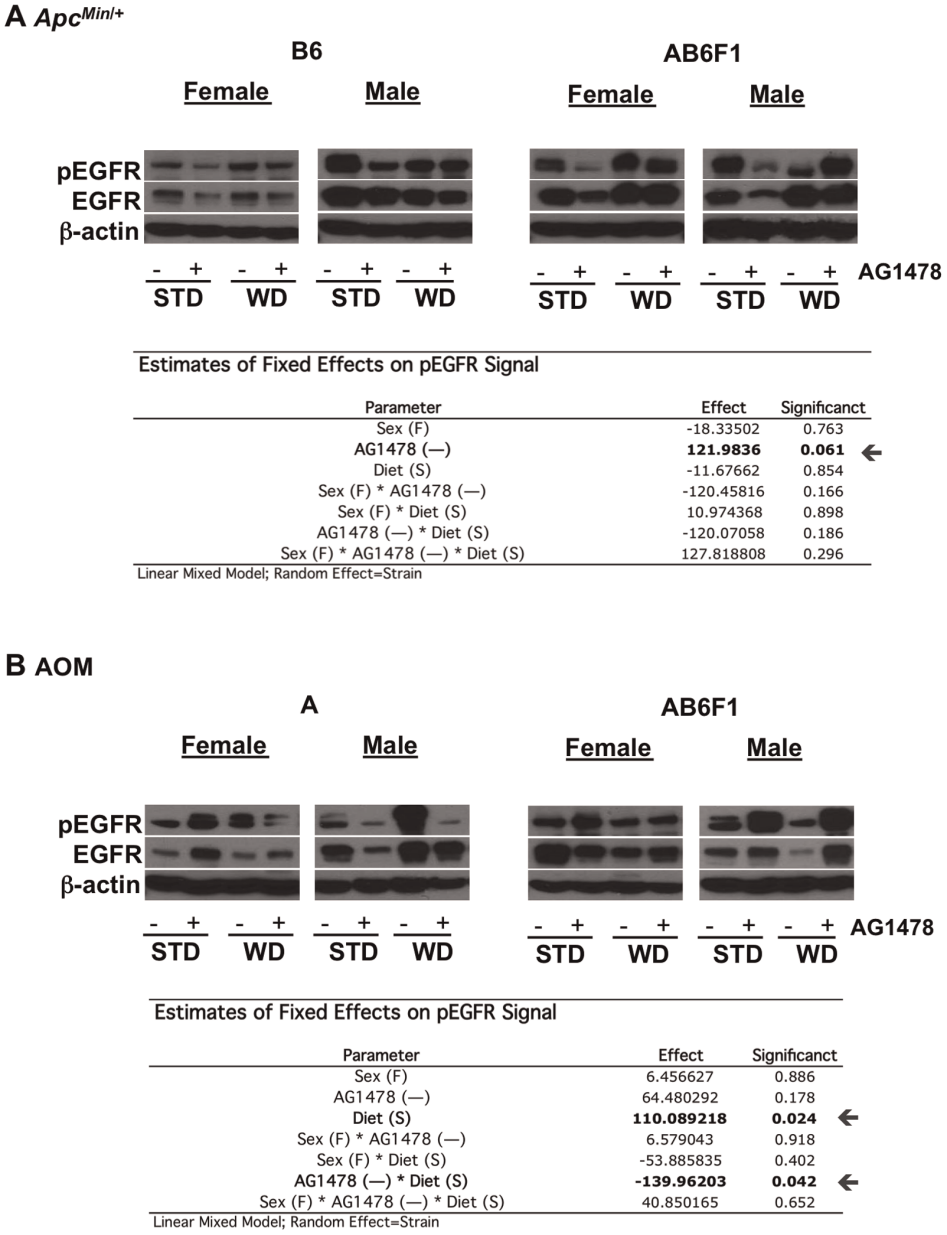
Western blot analysis of EGFR signal. Total EGFR and pEGFR in liver protein lysates from A) APC*^Min^* ^/+^ and B) AOM mice, separately for each strain and sex. STD = standard diet, WD = western diet, “+”  =  AG1478 treatment and “−“  =  no AG1478 treatment. Below bar graphs are estimated effects and significance based on linear mixed models.

For *Apc^Min/+^* mouse liver lysates linear mixed model results show that AG1478 is moderately associated with decreased pEGFR signal (Figure 4A, p = 0.061). Conversely, AG1478 alone does not affect pEGFR signal in AOM mouse liver lysates. However, when combined with diet AG1478 treatment is associated with increased pEGFR signal (Figure 4B, p = 0.042). STD alone is also associated with increased pEGFR signal compared to WD in AOM mice (Figure 4B, p = 0.024).

## Discussion

Designing preclinical studies for cancer therapeutics that more closely model conditions faced in human clinical trials is essential to improve predictions of drug efficacy. In the current study we investigated the efficacy of the small molecule EGFR inhibitor AG1478 for treatment of CRC using two mouse models, each on a distinct inbred strain and a common F1 genetic background to model genetic heterogeneity of the CRC patient population. Additionally, a standard mouse diet similar to that used in most preclinical studies was compared to the impact of modeling a Western-style diet on efficacy of EGFR inhibitor therapy.

We observed a different, sometimes dramatic, response to AG1478 for each of the four combinations of model and genetic background. Within the inbred strains, AG1478 significantly inhibits tumor number when combined with the STD in the B6-*Apc^Min/+^* model or with the WD in A mice treated with AOM. In the AB6F1 background, AG1478 reduces tumor number on both diets in the *Apc^Min/+^* model, but has little impact on tumor number on either diet in the AOM model, only trending towards an effect on STD. Whether using an inbred or F1 hybrid background, we essentially observed opposing results between the two models. In some cases where tumor number reduction was not observed, reduction in tumor size was observed suggesting that EGFR inhibition does have some efficacy even when tumor number is unaffected.

We also observed a differential response to diet alone. WD increased tumor number and size in some mice. Studies have shown that the EGFR pathway is influenced by many diet-related factors, including dietary and herbal supplements [22] and fatty acids [23], [24]. Unsaturated fatty acids activate EGFR signaling in a rodent model of breast cancer as well as human endothelial cells. In the rodent model, a diet high in polyunsaturated fat (corn oil) increased phospho-EGFR signal while monounsaturated fat (olive oil) decreased signal. In endothelial cells unsaturated fat in the form of oleic acid and polyunsaturated fatty acids induced EGFR activity and downstream MAPK activation, possibly through direct interaction with the receptor, and this induction depended on degree of saturation.

Since it is apparent that genetic background alone may not account for the contradictory response to AG1478 other factors should be considered. The B6-*Apc^Min/+^* and AOM-treated A mouse models also have differential susceptibility to diet-induced obesity with the B6 background being susceptible, while the A background is resistant. Furthermore, the distribution of tumors (entire intestinal tract versus colon only), age at onset of diet treatment (3 weeks versus 6 months), length of diet treatment (9 versus 12 weeks), initiating mutational events (*Apc* versus *Ctnnb1*) and potentially microflora may also contribute to model-dependent differences in response to AG1478. Since mice of the same model/strain were maintained within the same room we do not expect a significance difference in microflora within these groups but differences between the groups cannot be ruled out. Each of these differences, particularly susceptibility to obesity, distribution of tumors and microflora, likely contribute to strain and model differences in metabolism of dietary and therapeutic agents.

Finally, sex-dependent influences on tumor incidence and tumor response to both diet and AG1478 treatment exist. Overall tumor number was higher in AOM-treated female mice on A and AB6F1 backgrounds. In addition, only females were vulnerable to WD-induced tumors in the AOM model (data not shown). For this reason AG1478 impact on EGFR signaling was stratified according to sex in addition to model and strain.

In liver the impact of AG1478 on EGFR signaling is variable depending on model and diet. AG1478 is associated with decreased pEGFR signal in *Apc^Min/+^*mice. This is in agreement with the overall greater affect of AG1478 towards inhibiting tumor growth in this model. In AOM mice, though, diet is more strongly associated with pEGFR signal and AG1478 only affects pEGFR signal when combined with diet in the linear model.

Circumstances where AG1478 reduces EGFR signal but not tumor growth may indicate the presence of EGFR-independent tumors. We also must consider that, while AG1478 is metabolized in the liver, EGFR signaling in liver may not always reflect an equivalent impact on EGFR signaling in tumors. Additionally, dietary components or crosstalk with other pathways may interfere with AG1478 efficacy or our ability to observe its impact on signaling. Finally, we may be limited by the number of replicates, and closer observation of downstream signaling may be required.

Results for AG1478, which models a typical preclinical therapeutic study, demonstrates that better experimental designs are essential to improve the value of preclinical studies for predicting future clinical utility of new cancer therapeutics. We therefore propose that genetic background, sex, diet, and disease model be considered in future preclinical cancer studies. While initially these improvements may be more costly and time consuming, ultimately they will greatly reduce time and expense by more accurately informing human clinical trials.
